# Association of Serum Polyunsaturated Fatty Acids (PUFAs) and Children’s Dietary Inflammatory Index (C-DII^TM^) with Recurrent Respiratory Infections in Children: A Cross-Sectional Study

**DOI:** 10.3390/nu17010153

**Published:** 2024-12-31

**Authors:** Daiva Gorczyca, Karolina Szeremeta, Mariola Paściak, Bogumiła Szponar, Longgang Zhao, Michael D. Wirth, James R. Hebert, Natalia Związek, Anna Prescha

**Affiliations:** 1 Charité—Universitätsmedizin Berlin, Corporate Member of Freie Universität Berlin and Humboldt-Universität Berlin zu Berlin, Center for Chronically Sick Children Berlin, Augustenburger Platz 1, 13353 Berlin, Germany; 2 The Faculty of Medicine, Wroclaw Medical University, Pasteura 1, 50-367 Wroclaw, Poland; karolina.szeremeta@10g.pl; 3 Non-Public Health Care Facility ”Evita”, Spacerowa 15a, 57-402 Nowa Ruda, Poland; 4 Hirszfeld Institute of Immunology and Experimental Therapy, Polish Academy of Sciences, Rudolfa Weigla 12, 53-114 Wroclaw, Poland; mariola.pasciak@hirszfeld.pl (M.P.); bogumila.szponar@hirszfeld.pl (B.S.); 5 Cancer Prevention and Control Program and Department of Epidemiology and Biostatistics, Arnold School of Public Health, University of South Carolina, 915 Greene Street, Suite, Columbia, SC 29208, USA; lz7@email.sc.edu (L.Z.); wirthm@email.sc.edu (M.D.W.); jhebert@chi-llc.net (J.R.H.); 6 Brigham and Women’s Hospital and Harvard Medical School, 181 Longwood Avenue, Rm 450C, Boston, MA 02115, USA; 7 College of Nursing, University of South Carolina, 1601 Greene Street, Rm 607, Columbia, SC 29208, USA; 8 Department of Nutrition, Connecting Health Innovations, LLC., 1417 Gregg Street, Columbia, SC 29201, USA; 9 Department of Dietetics and Bromatology, Wroclaw Medical University, Borowska 211, 50-556 Wrocław, Poland; nataliaazwiazek@gmail.com

**Keywords:** polyunsaturated fatty acids, children’s dietary inflammatory index, diet, children, recurrent respiratory infections

## Abstract

To assess the associations between serum and dietary polyunsaturated fatty acids (PUFAs), as well as the inflammatory potential of diet measured by the Children’s Dietary Inflammatory Index (C-DII^TM^), and recurrent respiratory infections (RRIs) in children. We enrolled 44 children aged 3–16 years with RRIs and 44 healthy controls. Dietary intake was assessed using a 7-day food record from which PUFA intake and C-DII^TM^ were calculated. Serum PUFA levels were determined using gas–liquid chromatography–mass spectrometry. The dietary assessment showed a significantly lower fiber intake in children with RRIs. The RRI group had a higher inflammatory potential in the diet than healthy controls. Children with RRIs have higher serum levels of linoleic, arachidonic, and eicosapentaenoic acids than healthy subjects. A pro-inflammatory diet was positively associated with serum *n*-6 PUFA levels in both the groups. A high inflammatory potential of diet, body mass index over 75 percentile, and short breastfeeding duration were identified as risk factors for RRIs. The inflammatory potential of diet is strongly associated with RRIs in children and related to their PUFA status. Promoting breastfeeding and healthy dietary habits during childhood are crucial for implementing effective preventive management strategies.

## 1. Introduction

Respiratory tract infections are the most common cause of pediatric morbidity worldwide. In developing countries, recurrent respiratory infections (RRIs) are the leading cause of childhood mortality, resulting in over two million deaths annually [1]. Children with RRIs often require frequent healthcare services, treatment with antibiotics, and surgical procedures, and are more prone to asthma development in early life [2]. Moreover, RRIs harm the socio-economic sector, leading to absences from daycare or school, and are associated with difficulties in the child’s social integration and parental absence from work [2].

Although RRIs are rarely associated with true primary immunodeficiency, this is likely related to the immaturity of the immune system, particularly in the first years of life, when humoral and phagocytic immunity is not yet capable of combatting infectious agents [3]. While appropriate reference values obtained from environmental and genetic risk factors predisposing children to RRIs have been available for some time, and studies concerning better prevention and control of infections remain limited. It is postulated that diet may play an important role among the modifiable risk factors [4].

Optimal nutritional status is important to support immune reactivity [5]. Several reports have highlighted the beneficial effects of polyunsaturated fatty acids (PUFAs) in the prevention or treatment of various health conditions in children, including inflammation [6]. Wang et al. found differences in the PUFA blood profile in children with RRIs in comparison with healthy subjects [7]. Although the role of dietary *n*-3 PUFA supplementation in the occurrence of respiratory infections was examined in only a few studies, there has been observed a beneficial effect of supplementation on upper respiratory infections in the first year of life [8] and in older children [9]. Consistent with this evidence, other studies have demonstrated that the Mediterranean diet—characterized by high consumption of fresh, natural products such as fruits, vegetables, whole grains, pulses, and sources of unsaturated fatty acids, including extra virgin olive oil and nuts—may significantly contribute to symptom reduction in children with recurrent colds and frequent inflammatory complications [10].

However, disentangling the effect of a single nutrient for other nutrients is very difficult, given the wide range of interactions between nutrients. Along with a plethora of data supporting the role of dietary components in inflammatory and immune processes, tools have been developed to quantify the overall effect of nutrition on the inflammatory response. The Dietary Inflammatory Index (DII^®^) is a scoring algorithm for quantifying the inflammatory effect of diet on inflammation [11]. More recently, a corresponding dietary inflammatory index for children (C-DII^TM^) has been derived from the DII^®^ via the use of a database on pediatric population-based food consumption [12]. The DII^®^, or one of its variants, including the C-DII^TM^, has now been validated with inflammatory biomarkers in over 50 studies worldwide. Previous studies involving children and adolescents have reported associations between C-DII^®^ scores and cardiovascular disease, obesity, and asthma [12]. To date, the relationship between RRIs in children and the C-DII^TM^ has not been investigated.

The objective of this study was to investigate the association between the serum profile of PUFAs and the inflammatory potential of diet measured by the C-DII^TM^ in children with RRIs when compared with healthy children without RRIs. The secondary aim was to identify the diet-related risk factors for RRIs.

## 2. Materials and Methods

### 2.1. Study Groups

This single-center study included eighty-eight children of both sexes, born as full-term infants. The sample size calculation, taking into account the prevalence of RRI in European countries and the population of children aged 3–17 years in Poland, according to 2021 data from the Central Statistical Office (GUS), confirmed that the number of study participants is adequate (minimum of 88 individuals). The study was conducted between August 2011 and March 2012. The participants were divided into two groups.

The RRI group consisted of 44 children aged 3–16 years who visited the Clinic of Pediatric Immunology and Rheumatology at Wroclaw Medical University. These visits were made as part of day-patient family doctor appointments for diagnostic tests. To be included in the RRI group, a child had to meet at least one of the following criteria [13]:

(1) Have suffered more than six respiratory infections per year.
(2) Have suffered more than one respiratory infection per month involving the upper airways between September and April.
(3) Have suffered more than three respiratory infections per year involving the lower airways.

Children diagnosed with primary or secondary immunodeficiency disease were excluded from the RRI group.

The control group consisted of 44 healthy children aged 2.5–17 years who visited an outpatient department for a routine medical follow-up. Medical history was obtained, and physical examination was performed to ensure the child was healthy and in good physical condition.

Exclusion criteria for both groups included any of the following:

- Systemic diseases or chronic inflammatory diseases.
- History of any illness that affected growth.
- Acute febrile illness based on medical history and clinical examination.
- Family history of immunologic defects.
- Currently breastfeeding.
- Intake of PUFA-containing supplements.

The study protocol was approved by the Ethics Committee of Wroclaw Medical University.

Written consent was obtained from the parents or legal guardians of the participants, and from participants over 16 years of age, before participation in the study.

### 2.2. Study Design

Eligible participants underwent interviews and medical examinations by experienced physicians during their visit to the one-day clinic or outpatient department. The number and types of respiratory infections per year were determined from medical records and medical history, along with data on the child’s breastfeeding duration. Parents or legal guardians of all participants (from both groups) completed the questionnaire with warning signs of immunodeficiency adapted from an expert committee of the International Union of Immunological Societies [14].

Height and weight were measured by the same person before breakfast using standardized methods and equipment. To evaluate the nutritional status of the children in a clinical setting, body weight, height, and body mass index (BMI) percentiles were used and the results were compared with the relevant percentile curves [15]. The assessment categorized children as follows: within the 25th–75th percentile range (indicating normal growth), below the 25th percentile (indicating deficient body mass or short stature), or above the 75th percentile (indicating excess weight or height) [15].

### 2.3. Dietary Assessment

The dietary intake of each study participant was analyzed using a 7-day dietary record (5 weekdays and 2 weekend days). To minimize the seasonal influence on nutrient intake, the number of participants in both study groups was matched according to the season of the year. Parents were instructed to record all food and beverages consumed at home. They were asked to obtain information on the type and quantity of products consumed by the child from the person who held custody of the child in their absence. These food records were verified by a trained dietician and analyzed using the computer program Dieta 6.0 (National Food and Nutrition Institute, Warsaw, Poland). Intake of macronutrients and the most critical micronutrients (Ca, Fe, vitamin A and C) and the respective percentages of recommendations for Polish children were calculated [16]. Data associated with physical activity (excluding children less than 7 years old) and the intake of dietary supplements were also collected. Dietary data extracted from 7-day dietary records were used to calculate C-DII^TM^ scores for all participants, according to Khan et al. [12]. An inflammatory score was assigned to 27 dietary components based on its literature-derived effects on various inflammatory markers according to Shivappa et al. [11]. The nutrients included in the calculation of C-DII^TM^ were as follows (numbers in brackets refer to the nutrient unit and its overall inflammatory effect score, respectively): energy (kcal; 0.180), protein (g; 0.021), carbohydrates (g; 0.097), total fat (g; 0.298), fiber (g; −0.663), vitamins A (µg of retinol equivalent (RE); −0.401), B1 (mg; −0.098), B2 (mg; −0.068), B3 (mg; −0.246), B6 (mg; −0.365), B12 (μg; 0.106), C (mg; −0.424), D (µg; −0.446), E (mg; −0.419), and folate (µg; −0.190), saturated fatty acids (SFA) (g; 0.373), monounsaturated fatty acids (MUFAs) (g; −0.009), PUFAs (g; −0.337), *n*-3 fatty acids (g; −0.436), *n*-6 fatty acids (g; −0.159), β-carotene (µg; −0.584), cholesterol (mg; 0.110), caffeine (mg; −0.110), alcohol (g; −0.278), iron (mg; 0.032), magnesium (mg; −0.484), and zinc (mg; −0.313). The intake of these dietary components was compared with a children population-based food consumption database with Z-scores standardized per 1000 kcal, the overall C-DII^TM^ score was then calculated for each participant.

### 2.4. Laboratory Analyses

Venous blood samples for laboratory analyses were drawn in the early morning between 08:00 and 09:00 after overnight fasting. Whole blood samples were used for functional and cytometric tests on the day of blood collection (only in RRI group). Serum separated from blood samples was stored in Eppendorf test tubes at a temperature of −70 °C until measurements were obtained of serum immunoglobulin and PUFA concentrations.

#### 2.4.1. Immunological Parameters

The following measurements were performed for the RRI group, as follows: complete blood count (CBC); subpopulations of lymphocytes CD3+, CD3+CD4+, CD3+CD8+, CD3–CD56+, and CD19+; the phagocytic activity (phagocytic index) of polymorphonuclear (PMN) leukocytes in the blood; complement hemolytic activity of human serum (CH50) and immunoglobulins concentrations—IgG, IgA, and IgM—in the serum. When medical history revealed the possibility of allergy, the total IgE level was assessed. Serum immunoglobulin concentrations were measured by the turbidimetry method [17]; lymphocyte phenotypes were identified by using the Beckman EPICS XL-MCL flow cytometer (Beckman Coulter Inc., Brea, CA, USA) [18]; the phagocytic index was defined as the mean values of Staphylococcus aureus 209P bacteria ingested per 100 PMN cells [19]; and the complement hemolytic activity of human serum against IgG-opsonized sheep red blood cells (the intensity of hemolysis) [20] was estimated on a spectrophotometer (DYNATECH MR 5000, Dynex Technologies, Chantilly, VA, USA) at λ = 540 nm and quantitatively calculated according to the standard hemolytic curve.

#### 2.4.2. PUFA Concentrations in Serum

Serum PUFA concentrations were determined in both RRI and control groups using a method described elsewhere [21]. Briefly, lipids were extracted from serum samples (0.2 mL) with a hexane–ethanol mixture, then an organic phase was collected and evaporated under nitrogen. After adding methanol solutions of internal standard (C23:0) and butylated hydroxytoluene (BHT) as an antioxidant agent, total fatty acids in samples (including both free and phospholipid-bound fractions) were converted to the corresponding fatty acid methyl esters (FAMEs) via the addition of methanol and acetyl chloride at 85 °C for 3 h. The FAMEs were extracted twice using hexane and then concentrated by evaporation under nitrogen. The obtained FAMEs were analyzed by gas chromatography coupled with mass spectrometry (Quantum TSQ apparatus, Thermo Scientific, Austin, TX, USA) on a Zebron ZB-5MS 30 m × 0.25 mm column (Phenomenex, Torrance, CA, USA), in the temperature program of 100 °C–175 °C, 25 °C/min, and then 175 °C–235 °C, 4 °C/min. The injector and transfer line between GC and MS were kept at 280 °C. The raw mass spectrometry data were processed using Thermo Xcalibur 2.0.7 software. FAMEs were identified by comparison with the standards and corresponding mass spectra and quantified with help of internal standard.

### 2.5. Statistical Analyses

Statistical analyses were performed using Statistica 13.0 (StatSoft, Krakow, Poland). Differences between the RRI and control groups were assessed using Student’s *t*-test, Mann–Whitney U test, and χ^2^ test, depending on the type of variables and their distribution. Correlations between the dietary variables and serum concentrations of PUFAs were calculated using the Pearson coefficient with Box–Cox transformation for variables that were not normally distributed. The odds of RRIs were calculated in a multivariable-adjusted model using R-4.3.1 and with *p*-values < 0.05. In the case of statistically significant test results, the post-hoc power analysis was performed using G*POWER 3.1 software. A receiver operating characteristic (ROC) discriminant analysis was performed for the strongest predictor identified in the multiple regression analysis. To enhance the robustness of the analysis, 1000 bootstrap samples were employed to estimate confidence intervals (CIs) for the area under curve (AUC) and cut-off point, ensuring reliability of the results.

## 3. Results

### 3.1. Characteristics of the Groups

The characteristics of the study groups are presented in Table 1. The groups were similar in terms of age, sex, weight, and height. Upon grouping based on BMI and breastfeeding duration, higher percentages of children above the 75th percentile BMI and breastfeeding below 6 months were present in the RRI group than in the control group (*p* = 0.0068 and *p* = 0.033, respectively). The number of siblings were also differently distributed in the RRI and control groups (*p* = 0.0001). Immunological parameters for which the reference ranges differ according to the child’s age are shown in Table 1 as the number and percentage of participants with values not within the reference range. Serum IgA concentrations lower than the reference range were observed in five children (12%) with RRIs, serum total IgE levels above the reference range were observed in eight children (33%), and eosinophilia was observed in six children (14%) (Table 1). All children with RRIs and controls had serum IgG and IgM levels and subpopulations of CD3+, CD3+CD4+, CD3+CD8+, CD3–56+, and CD19+ within the reference range. Phagocytic index and CH50 did not differ significantly between the RRI and the control group.

### 3.2. Analysis of Diet

The analysis of the children’s diets focused on evaluating the C-DII^TM^ values and the intakes of macronutrients, critical micronutrients, and fatty acids, along with the percentage of corresponding recommendations, when applicable (Table 2). The intake of energy and energy components (carbohydrates, fat and protein) did not differ between groups. There were also no significant differences in fat composition, either in terms of individual PUFA intakes or the contribution to energy supply by fatty acid groups (SFA, MUFA and PUFA). The contribution of linoleic acid (LA) to the energy of the diet of children in the RRI and control groups was below the recommended 4%. Moreover, VLC-PUFA intake (EPA + DHA) was very low, accounting for 15.3 and 20.4% of the recommended AI in the RRI and control groups, respectively. The ratio of *n*-6/*n*-3 fatty acids in the diet was similar in both groups and, at ca. 6. The diet of children with RRIs provided significantly less fiber (7.97 g/1000 kcal vs. 9.43 g/1000 kcal, *p* = 0.0039) and vitamin A (98.2%RDA vs. 131.5%RDA, *p* = 0.039) compared with the control group. Moreover, the diet of children with RRIs had a higher proportion of pro-inflammatory components, as indicated by higher C-DII^TM^ scores (0.26 vs. −0.92, *p* < 0.0001).

### 3.3. Serum PUFA Profile

Serum levels of linoleic acid (LA), arachidonic acid (AA), and eicosapentaenoic acid (EPA) were significantly higher in the RRI group than in the control group (*p* = 0.0085, *p* = 0.014, and *p* = 0.0009, respectively) (Table 3).

### 3.4. The Relation of Dietary Parameters to Serum PUFA and the Impact of Dietary Related Factors on RRI Risk

Table 4 presents significant Pearson correlations between dietary factors and serum PUFA levels after adjustment for selected confounders, including sex, age, BMI percentile range, and time of breastfeeding in the past. In the RRI group, the energy intake from LA and total PUFAs was positively related to serum ALA concentration (r = 0.43 and 0.42, respectively, *p* ≤ 0.005). Dietary ALA and total PUFAs were inversely associated with PUFA *n*-6/*n*-3 ratio in serum in this group (r = −0.36 and −0.32, respectively, *p* ≤ 0.046). Moreover, a positive correlation was observed between serum AA level and energy-adjusted fat intake (r = 0.42, *p* = 0.007) and C-DII^TM^ (r = 0.55, *p* = 0.000), while a negative correlation was found with LC-PUFAs (r = −0.33, *p* = 0.040) and fiber intake (r = −0.48, *p* = 0.002). Fiber intake also showed a moderate positive correlation with serum DHA (r = 0.43, *p* = 0.005) and a negative correlation with the serum *n*-6/*n*-3 PUFA ratio (r = 0.49, *p* = 0.001). In the control group, energy intake and C-DII^TM^ score were positively associated with serum LA (r = 0.33 and 0.35, respectively, *p* ≤ 0.035), while, for energy intake from PUFAs, a negative correlation was found with LA level in serum (r = −0.36, *p* = 0.022). C-DII^TM^ was also positively related to the serum *n*-6/*n*-3 PUFA ratio (r = 0.41, *p* = 0.008); an opposite association between fiber intake and serum AA was observed in healthy subjects (r = −0.36, *p* = 0.024).

### 3.5. Estimation of the RRI Risk Associated with the Studied Parameters

**Figure 1:**
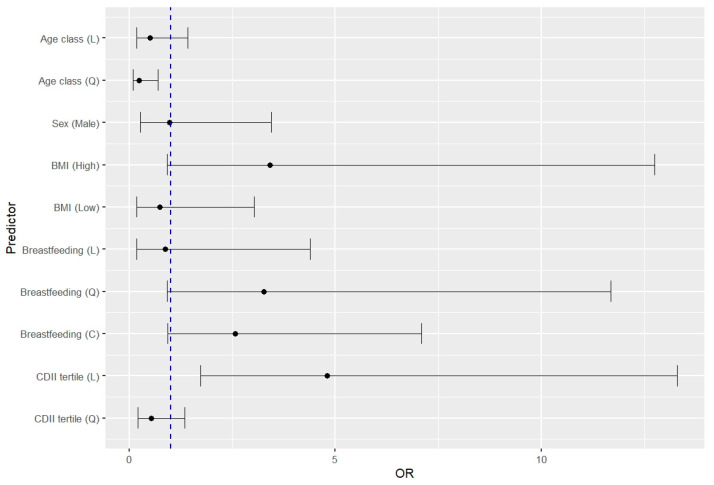
Forest plot of multiple regression model with selected predictors of respiratory tract infections (RRIs). The dotted line represents the null effect. Due to the failure to meet the linearity condition, the following ordinal variables were used: age (1 = <6 y; 2 = 6–12 y; 3 = >12 y), breastfeeding (1 = >12 months; 2 = 7–12 months; 3 = 0–6 months; 4 = 0 months), children dietary inflammatory index (C-DII^TM^) (1 = 1st tertile; 2 = 2nd tertile; 3 = 3rd tertile). For percentile BMI (kg/m^2^) the 25–75th range was used as a reference, BMI (high) = <25th BMI percentile, BMI (low) = >75th BMI percentile. C-DII^TM^ tertiles: 1st from −4.11 to −1.01; 2nd from −0.94 to 0.29; 3rd from 0.39 to 3.32. L—linear component; C—quadratic component; Q—qubic component.

**Figure 2:**
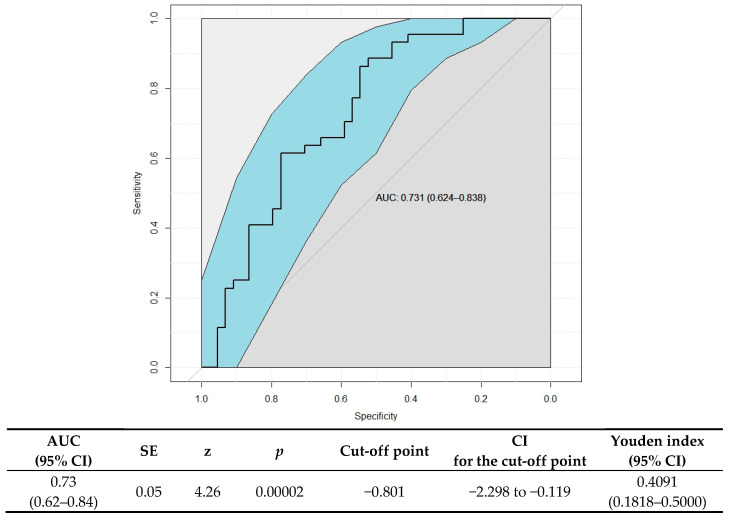
Receiver operating characteristic (ROC) curves for C-DII^TM^ as a predictor of RRI risk and its optimal cut-off point, determined using the Youden index. The blue color represents the 95% confidence bounds. AUC—area under curve; CI—confidence interval.

Medical history data and dietary estimates allowed us to identify the potential risk factors for RRIs in children. BMI above the 75th percentile (OR = 3.42, 95% CI 0.92–12.72) and an increase in the proinflammatory potential of the diet (OR = 4.80, 95% CI 1.73–13.30) were risk factors for RRIs (Figure 1). Moreover, the OR value markedly decreased as the children grew older. There was also a trend in increasing the risk of RRIs with a shorter duration of breastfeeding below 12 months, although this was only discernible for exponentiated data (OR = 3.27, 95% CI 0.91–11.68). For the C-DII™ parameter, identified as the strongest predictor in the multiple regression analysis, an ROC discriminant analysis was performed. The area under the ROC curve (AUC) was 0.73 (95% CI: 0.62–0.84), indicating moderate discriminative ability of this parameter in predicting RRI risk (Figure 2). The optimal cut-off point, determined using the Youden index, was −0.801, which balances sensitivity and specificity. The 95% confidence interval for the cut-off value, estimated using the bootstrap method with 1000 iterations, ranged from −2.298 to −0.119. Additionally, the Youden index value was 0.4091 (95% CI: 0.1818–0.5000), further supporting the utility of this cut-off point in differentiating RRI risk.

## 4. Discussion

To the best of our knowledge, this is the first study to focus on the association between the dietary inflammatory potential measured by C-DIITM scores and serum concentration of PUFAs in children with RRIs and the risk of RRIs in children.

It is worth emphasizing that, in our RRI group, one-third of the children exhibited elevated IgE levels and 14% had eosinophilia. Against this background, unrecognized allergic diseases, which can mimic RRIs in their clinical presentation, merit careful consideration. Furthermore, atopic disease is a strong determinant of all upper respiratory tract infections [22]. In this context, it is notable that Toivonen et al. observed asthma diagnosed in 12% of children with respiratory infections [2].

Analysis of the children’s diets revealed no differences in macronutrient intake or dietary fatty acid composition between healthy and RRI children. However, both groups were characterized by very low dietary VLC-PUFA intake, providing an average of no more than 20% of the recommended amounts of these nutrients [16]. This deficiency may be detrimental in RRI, as eicosanoids derived from the conversion of EPA and DHA, as well as AA, play a key role in balancing immune system reactivity [23,24]. The recently disclosed role of lipid mediators, including specialized pro-resolving mediators (SPMs), derived from both *n*-3 and *n*-6 PUFAs, is not limited to resolving inflammation but also includes supporting the host’s antimicrobial defenses during infections [25]. The beneficial effects of VLC-PUFA supplementation in respiratory infections have been demonstrated in several studies involving both adults and children [6,8,26,27]. Additionally, the total contribution of LA and ALA to energy supply in both RRI and control group was not at a satisfactory level and amounted to less than 4% and 0.5%, respectively [16]. Total PUFA intake has been demonstrated to have a beneficial effect on systemic inflammation in adults, measured as CRP concentration in blood. A similar relationship was shown for LA consumption [28]. LA, in addition to being a substrate for AA, may exert anti-inflammatory effects, e.g., via 13-HODE [23]. In contrast, according to recently published metanalysis, short- and long-term interventions with ALA supplementation has no significant effect on inflammatory biomarkers in adults [29].

The key distinguishing factors in the diets of children with RRIs were lower fiber intake and a higher C-DII^TM^ score (more pro-inflammatory diet) compared with the control group. Anti-inflammatory dietary components have already been linked to airway function in children and adults. Ingredients with demonstrated independent anti-inflammatory effects on the respiratory tract include fiber and vitamins A, C, and D [4,5], along with the aforementioned PUFAs, all of which are incorporated into the C-DII^TM^ score. Han et al. showed that a higher DII^®^ score is associated with elevated fractional exhaled nitric oxide (a marker of eosinophilic airway inflammation) and increased odds of current wheezing in children [30]. In our study we found a significant positive association between the C-DII^TM^ score and the risk of RRIs. The high inflammatory potential of the diet was associated with an almost 5-fold increase in disease risk. Moreover, the C-DII^TM^ parameter demonstrated a moderate discriminative ability (AUC = 0.73) in distinguishing children at risk of RRI, indicating its utility as a supportive marker in dietary assessment. The C-DII^TM^ cut-off point value of −0.801 suggests that dietary approach focusing on the provision of predominantly anti-inflammatory components may play a key role in preventing RRI. One of the components of C-DII^TM^ with a strong anti-inflammatory effect is dietary fiber. Interestingly, several studies have reported that patients with asthma tend to consume less fiber compared with healthy controls [31]. Most potential mechanisms of fiber in improving airway inflammation promote anti-inflammatory cytokine production and modulate the gut-related immune response through the fermentation products of soluble fiber (e.g., short chain fatty acids) [32]. The immunomodulatory effects of fermentable fiber, which includes pectin, resistant starch, gums, mucilage and oligosaccharide carbohydrates, are attributable to the modification of the microflora within the colon, as well as the direct and indirect effects on the intestinal epithelium and innate immune cells. The evidence for the preventive role of prebiotic fiber components in RRI has been published elsewhere [33,34,35]. It is suggested that the gut–lung axis manipulation of intestinal microflora may be helpful in the treatment of lung diseases [36]. The microflora can activate the pulmonary biota via the granulocyte-macrophage colony-stimulating factor (GM-CSF) signal pathway, and thereby enhance the resistance to respiratory tract infections [37]. In light of existing studies and the results of this research, ensuring an adequate dietary intake of fiber, particularly its fermentable fractions, appears to be of paramount importance for children with RRI. Unfortunately, the present study was unable to determine the proportion of soluble dietary fiber consumed by the participants, as this level of detail is not provided by the Polish product database used in the Diet 6.0 software. It should be mentioned, however, that some types of insoluble fiber, such as mannan and, to a lesser extent, cellulose, also possess fermentable properties and can influence the gut microbiota and immune function [38]. Given these considerations, further investigation into the role of fiber intake in RRI risk is warranted. Future research should evaluate the dietary patterns that could help capture the participation of fiber-rich food intake, such as fruits, vegetables, legumes and cereals, in the prevention and management of RRI.

In the current study, we found higher serum levels of LA, AA, and EPA in children with RRIs than in the healthy controls. The observed differences in serum PUFA profiles in children with RRI may have significant implications due to the pivotal role of PUFAs as precursors of lipid signaling molecules involved in numerous biological processes, including the mediation of inflammatory responses. This encompasses both pro- and anti-inflammatory eicosanoids, hydroxy-, hydroperoxy-, epoxy-, oxo-eicosanoids, as well as SPMs [39]. Higher serum LA and AA concentrations in children with RRI may indicate greater direct and indirect substrate availability for the production of mostly pro-inflammatory eicosanoids, such as prostaglandins, leukotrienes, and lipoxins, by cyclooxygenases and lipooxygenases. AA can also form lipoxins, LXA4 and LXB4, that are considered part of a group of SPMs. A change in AA metabolism from synthesis of leukotrienes to lipoxin formation occurs in the early phase of inflammation. Subsequently, SPM production results in the mobilization of *n*-3 VLC-PUFA, which are substrates for protectins, resolvins and maresins [39]. It is worth noting that there are a number of important signaling molecules in humans, which are formed directly from LA or ALA (e.g., octadecanoids) [40]. Moreover, the availability of PUFAs for further metabolism, and their involvement in inflammatory processes, is facilitated by the action of phospholipase A2, which releases PUFAs from phospholipid membranes and this process may be enhanced by various factors, such as hormones, but also by non-specific pathological conditions [41]. The differences in serum fatty acid profiles observed in this study between children with RRI and healthy controls suggest that the inflammatory milieu and immune response mediated by lipid-derived mediators may be altered in affected children; however, the underlying mechanisms of this phenomenon require further detailed investigation. There are only limited studies that have explored the association between serum PUFA levels and infections in children. In the multinational study, a negative relationship was found between serum EPA and DHA level at birth and Coxackievirus B2 infection during the first 18 months of life [42]. Serum DHA and AA was positively associated with influenza A IgG levels among influenza A seropositive children, and, moreover, LA level was linked to the number of Coxsackievirus B (CVB) infections. Interestingly, Wang et al. have observed lower levels of LA in viral infection compared with bacterial infection in febrile children, suggesting enhanced lipogenesis and fatty acid uptake in the host cell during viral replication [7]. Several studies on PUFA composition in blood in children with allergy yielded inconsistent results, including increased LA levels but decreased AA levels in buffy coat cells [43], increased levels of both LA and AA in plasma [44] and elevated EPA levels in both plasma and cell membranes [45]. The findings from the latter two studies partially align with our results, contributing to the understanding of altered PUFA metabolism in children with RRIs and allergies.

In our study, no clear correlations were observed between the dietary intake of PUFAs and their serum levels in either the RRI or control groups. Similar findings have been reported in studies involving healthy children and obese pubertal children with metabolic syndrome [46]. Several previous studies have suggested that the concentration of fatty acids in the blood reflects an individual’s endogenous metabolism rather than their dietary intake [47]. In our study, we employed a 7-day dietary record method to assess intake, which is considered the gold standard in dietary assessment due to its demonstrated strong correlation with biomarkers of dietary intake for exogenous fatty acids, comparable to semiquantitative FFQs. However, the strongest association with PUFA serum levels appears to be achieved using an average derived from repeated seven-day recalls conducted months apart [48]. Moreover, the type of study (short- or long-term), metabolic characteristics, and expected variability in fatty acids are crucial for determining which tissues best reflect true intake. Serum cholesterol esters are the most suitable for short-term dietary compliance, though caution is needed due to multiple influencing factors. However, serum, serum fractions, and erythrocyte membranes may also reflect short-term intake of fatty acids (days to months) and are commonly used in observational studies due to their accessibility and the assumption of stable short-term diets [49]. The weak influence of fatty acid intake on the serum PUFA profile observed in both groups may also stem from significant deficiencies in dietary polyunsaturated fatty acids, particularly VLC-PUFA *n*-3, as well as a lower-than-recommended energy contribution from ALA and LA [50]. Moreover, since this study did not assess inflammatory markers, we are unable to determine whether the changes in PUFA profiles were influenced by levels of inflammatory markers. The relationship between dietary and serum fatty acids and inflammation has been extensively studied, revealing that different types of fatty acids can have varying effects on inflammatory processes [51]. As mentioned earlier in this work, while some hypotheses suggest that high intake of *n*-6 PUFAs may promote inflammation, recent evidence indicates that adequate consumption is associated with better cardiovascular health and child development, without necessarily increasing systemic inflammation [28,52]. Serum levels of total and individual PUFAs in healthy adults have been shown to correlate with decreased levels of inflammatory markers. The strongest associations were observed between AA and reduced interleukin-6 levels, and between DHA and decreased TNF-α levels, whereas EPA concentration had the weakest impact on inflammatory markers [53].

Among dietary components, the strongest association was found for fiber intake, which exhibited a negative correlation with serum AA levels in both groups and a positive correlation with DHA levels in children with RRIs. We have previously demonstrated that children with allergies had lower serum levels of LA than healthy children who followed a vegetarian diet [21]. A vegetarian diet rich in fiber (particularly the fermentable fraction of fiber) is associated with various beneficial metabolic effects. Interestingly, a study conducted on mice demonstrated that the consumption of modified soluble legume fiber resulted in a reduction in blood levels of *n*-6 PUFA in mice fed a high-fat diet, along with changes in other serum metabolic properties [54].

It should be noted that our study is the first to reveal the relationship between C-DII^TM^ and PUFA profile in the blood. An important finding is the positive relationship between higher C-DII^TM^ scores (indicating a pro-inflammatory diet) and serum AA levels in children with RRIs. At the same time, in healthy subjects, C-DII^TM^ was found to be positively related to serum *n*-6/*n*-3 PUFA ratio. The theoretical background of the “linoleic acid pro-inflammatory paradigm” was built on an enhanced synthesis of pro-inflammatory eicosanoids derived from AA and diminished synthesis of anti-inflammatory eicosanoids from EPA and DHA. However, an increasing amount of evidence from human studies suggests that *n*-6 PUFAs also provide some anti-inflammatory activity, similar to *n*-3 PUFAs [55]. Considering the results of our study, we assume that the PUFA metabolism, may be disturbed in RRI. Moreover, the PUFA metabolism is influenced by selected nutrients and their metabolites in a complex manner. Overall, our results, along with those from previous studies [21,56], align with existing evidence concerning the associations between diet and immune conditions.

An additional conclusion of this study concerns the diet-related risk factors of RRIs in children. We found that an increased risk of RRIs was associated with a BMI above the 75th percentile, consumption of a high pro-inflammatory diet, and a shortened duration of breastfeeding. There is substantial evidence suggesting that breastfeeding may reduce the risk of overweight in childhood. Research shows that children who are breastfed have a 22% lower risk of childhood obesity compared with those who were never breastfed. Furthermore, breastfeeding during the first months of life has been linked to a reduced risk of childhood obesity, independent of maternal body weight [57]. While breastfeeding appears to have a positive effect on reducing the risk of overweight and obesity, its impact on the risk of underweight remains inconclusive [58]. In our study, we did not observe an association between the duration of breastfeeding and the BMI of the children. This allowed us to consider both factors as independent predictors in the multiple regression model. Similar to our results, other studies have shown an association between breastfeeding duration of up to 6 months and an increased risk of RRIs [59]. Previous investigations have also demonstrated that children who ate a high pro-inflammatory diet had higher BMI and were more likely to reveal higher levels of inflammatory cytokines [60]. Moreover, consumption of a pro-inflammatory diet during childhood is associated with asthma and increases the odds of current wheezing in adults and children with allergic (atopic) wheezing [32]. Our results support the hypothesis that a nutritionally adequate, anti-inflammatory diet in children and adolescents with a healthy weight is associated with a reduced risk of infection.

Despite its strengths, this study has several limitations. Although the sample size was relatively small, the study design enabled us to identify significant associations and provide a comprehensive picture of these interactions. The cross-sectional design of the study and absence of dietary interventions make causal inferences difficult. Thus, we were not able to conclude whether the presence of RRIs leads to a more pro-inflammatory diet or whether a pro-inflammatory diet leads to RRIs in children. Additionally, while a link between breastfeeding duration and body weight has been observed in the literature, it was not revealed in our study. This suggests the need for further investigation in a larger cohort to determine whether RRIs may alter this relationship. Furthermore, due to the significant role of fiber in the dietary influence on PUFA profiles, a more detailed assessment of diet is needed, particularly focusing on the intake of different fiber fractions. Nevertheless, this study provides a rationale for appropriate intervention studies.

## 5. Conclusions

In our study, we showed that the serum PUFA profile of children with RRIs was significantly different from that of healthy children, regardless of the absence of differences in dietary PUFA intake. Diet quality, particularly in terms of inflammatory potential, is strongly and positively associated with RRIs in children. The inflammatory potential of diet and nutritional status assessment may represent crucial factors in comprehensive interventions to combat RRIs as well as for establishing rational preventive dietary management.

## Figures and Tables

**Table 1:** Characteristics of children with RRI and healthy controls.

	RRI Group (*n* = 44)	Control Group (*n* = 44)	*p*-Value
Age (*y*), median (Q1–Q3)	6.5 (3.0–16.3)	6.3 (2.5–17.0)	0.49
Sex, female/male, *n* (%)/*n*(%)	20 (45)/24 (55)	20 (45)/24 (55)	0.99
Weight (percentile ranges), *n* (%)			
<25	5 (11)	6 (14)	0.58
25–75	21 (48)	25 (57)	
>75	18 (41)	13 (30)	
Height (percentile ranges), *n* (%)			
<25	6 (14)	3 (7)	0.57
25–75	25 (57)	26 (59)	
>75	13 (30)	15 (34)	
BMI (percentile ranges), *n* (%)			
<25	4 (9)	11 (25)	**0.0068**
25–75	16 (36)	23 (52)	
>75	24 (55)	10 (23)	
Breastfeeding (months), *n* (%)			
0	9 (20)	2 (5)	**0.033**
1–6	12 (9)	23 (52)	
7–12	17 (39)	14 (32)	
>12	6 (14)	5 (11)	
Subjects with IgA levels lower than the normal range depended on age, *n* (%)	5 (12)	0 (0)	…
Subjects with IgM and IgG levels lower than the normal range depended on age, *n* (%)	0 (0)	0 (0)	…
Subjects with IgE levels higher than the normal range depended on age, *n* (%)	8 (33) *	0 (0) **	…
Subjects with eosinophilia, *n* (%)	6 (14)	0 (0)	…
Subjects with CD3+, CD3+CD4+, CD3+CD8+, CD3–56+, and CD19+ levels lower than the normal range depended on age, *n* (%)	0 (0)	0 (0)	…
Phagocytic index, median (Q1–Q3)	3.42 (2.78–4.61)	3.38 (2.80–3.98)	0.716
CH50, median (Q1–Q3)	77.66 (55.52–92.08)	85.12 (70.46–119.83)	0.740

*—measured in 24 subjects; **—measured in 17 subjects. Differences with *p* < 0.05, in bold. Intergroup comparisons of continuous variables were performed using the Mann–Whitney U test, in case of categorical variables χ^2^ test was used.

**Table 2:** Dietary intakes and dietary fat composition with respective recommendations [16], and energy-adjusted children’s dietary inflammatory index (C-DII) in the RRI and control groups; median (Q1–Q3).

Dietary Parameter	Recommended Intake	RRI Group	Control Group	*p*-Value
Energy				
Intake, kcal % EER	(a)	1493 (1308–1831) 91.6 (70.3–123.8)	1397 (1261–1900) 92.7 (68.1–111.0)	0.728 0.210
Protein				
Intake, g/1000 kcal % RDA	(b)	38.3 (33.4–41.1) 235.2 (140.6–285.6)	37.8 (35.1–42.1) 277.1 (145.5–271.6)	0.966 0.299
Carbohydrates (available)				
Intake, g/1000 kcal RI (%)	45–65% energy	135.2 (123.6–146.5) 54.4 (49.8–58.9)	135.3 (122.9–142.6) 54.5 (49.5–57.4)	0.172 0.732
Fiber				
Intake, g/1000 kcal % AI	(b)	7.97 (6.75–8.90) 77.8 (70.2–94.3)	9.43 (7.41–10.91) 103.5 (75.1–116.3)	**0.004** 0.006 *
Fat				
Intake, g/1000 kcal % RDA	(a)	33.0 (28.4–35.5) 87.6 (67.5–113.6)	33.1 (28.0–38.4) 83.0 (43.4–156.4)	0.220 * 0.344
SFA, % energy	Lower than 10% energy (<10 y); 5–6% energy (≥10 y)	12.0 (9.6–13.3)	11.7 (10.5–13.7)	0.368
MUFA, % energy	-	10.7 (9.0–12.2)	10.3 (8.6–12.8)	0.160
PUFA, % energy	-	3.33 (2.52–4.25)	3.86 (2.70–4.81)	0.073
LA, % energy	4% energy	2.73 (2.17–3.55)	3.17 (2.27–3.87)	0.083 *
ALA, % energy	0.5% energy	0.42 (0.34–0.58)	0.45 (0.37–0.56)	0.394
AA intake, mg/day	-	54.5 (33.5–102.6)	55.4 (28.5–92.7)	0.730
EPA intake, mg/day	-	7.4 (3.27–16.71)	14.8 (3.97–35.7)	0.104
DHA intake, mg/day	-	26.4 (9.08–43.69)	33.3 (17.6–60.1)	0.224
VLC-PUFA, % AI	250 mg/day EPA + DHA	15.3 (6.6–24.6)	20.4 (11.4–46.6)	0.107
*n*-6/*n*-3 FA ratio	-	6.02 (4.64–7.30)	6.00 (4.71–7.62)	0.689
Vitamin A, µg/1000 kcal %RDA	(b)	353.8 (284.2–500.2) 103.2 (77.6–189.6)	511.3 (363.5–774.5) 138.1 (99.1–239.3)	**0.039**
Vitamin C, mg/1000 kcal %RDA	(b)	36.7 (18.3–46.8) 104.1 (65.9–128.7)	41.3 (23.6–66.9) 109.5 (73.2–196.3)	0.157
Calcium, mg/1000 kcal %RDA	(b)	366.7 (280.5–477.3) 56.5 (35.6–68.9)	364.1 (303.7–497.2) 57.3 (40.9–81.9)	0.486
Iron, mg/1000 kcal %RDA	(b)	4.61 (4.13–5.31) 71.0 (56.8–86.2)	5.06 (4.50–5.70) 82.9 (61.6–100.1)	0.104 *
C-DII	-	0.26 (0.95–2.53)	−0.92 (−1.30–1.66)	<0.001 *

Differences with *p* < 0.05, in bold. Intergroup comparisons were performed using the Mann–Whitney U test, *p*-values marked with an asterisk were calculated using the parametric Student’s *t*-test. EER—estimated energy requirements; RDA—recommended dietary allowance; RI—reference Intake ranges for macronutrients; AI—average intake; SFA—saturated fatty acids; MUFA—monounsaturated fatty acids; PUFA—polyunsaturated fatty acids; LA—linoleic acid; ALA—α-linolenic acid; AA—arachidonic acid; EPA—eicosapentaenoic acid; DHA—docosahexaenoic acid, VLC-PUFA—very long chain-PUFA, *n*-6/*n*-3 FA ratio—the ratio of fatty acids from *n*-6 to *n*-3 family; C-DII—energy-adjusted children’s dietary inflammatory index. (a)—age, sex and physical activity level (PAL) dependent. (b)—age and sex dependent.

**Table 3:** Serum PUFA in children with RRI and healthy controls (mg/dL).

Fatty Acid	RRI Group	Control Group	*p*-Value
LA	59.78 (41.51–79.11)	51.28 (36.00–78.23)	**0.025**
AA	12.62 (6.92–20.18)	10.59 (6.98–18.22)	**0.042**
ALA	1.57 (0.61–3.46)	1.89 (0.42–3.62)	0.911
EPA	1.74 (0.76–3.05)	1.30 (0.55–2.69)	**0.007**
DHA	2.76 (1.32–8.75)	2.38 (1.20–4.47)	0.113
*n*-6/*n*-3 FA ratio	12.52 (4.64–22.40)	12.52 (4.99–21.52)	0.841 *

Differences with *p* < 0.05, in bold. Intergroup comparisons were performed using the Mann–Whitney U test, *p*-value marked with an asterisk was calculated using the parametric Student’s *t*-test. LA—linoleic acid; ALA—α-linolenic acid; AA—arachidonic acid; EPA—eicosapentaenoic acid; DHA—docosahexaenoic acid; *n*-6/*n*-3 FA ratio—the ratio of fatty acids from the *n*-6 to *n*-3 family.

**Table 4:** Correlations of dietary fat components, C-DII^TM^, and fiber with the level of PUFAs in serum adjusted by age, sex, BMI percentile, and breastfeeding duration.

	Serum PUFAs					
	LA	AA	ALA	EPA	DHA	PUFA *n*-6/*n*-3
RRI group						
Fat, intake/1000 kcal	0.15 (0.35)	**0.39 (0.013, 0.784)**	0.16 (0.32)	0.07 (0.65)	−0.08 (0.62)	0.10 (0.56)
LA, % energy of diet	−0.08 (0.63)	−0.14 (0.40)	**0.42 (0.007, 0.851)**	0.12 (0.48)	0.11 (0.50)	−0.25 (0.12)
ALA, % energy of diet	−0.17 (0.31)	−0.15 (0.36)	0.09 (0.59)	0.12 (0.45)	**0.34 (0.032, 0.649)**	**−0.36 (0.03, 0.706)**
PUFAs, % energy of diet	−0.01 (0.94)	−0.20 (0.21)	**0.39 (0.0012, 0.784)**	0.11 (0.48)	0.18 (0.28)	**−0.32 (0.046, 0.591)**
*n*-3 PUFAs, % energy of diet	−0.01 (0.94)	−0.20 (0.21)	0.04 (0.82)	−0.10 (0.54)	**−0.34 (0.031, 0.561)**	−0.20 (0.22)
EPA, mg/day	−0.16 (0.32)	**−0.34 (0.032, 0.591)**	−0.12 (0.48)	−0.06 (0.74)	0.02 (0.91)	0.02 (0.89)
DHA, mg/day	−0.21 (0.14)	**−0.34 (0.031, 0.561)**	0.05 (0.75)	−0.18 (0.25)	0.14 (0.39)	−0.11 (0.50)
Fiber, intake/1000 kcal	−0.20 (0.21)	**−0.44 (0.004, 0.888)**	0.05 (0.77)	−0.04 (0.80)	**0.45 (0.005, 0.904)**	**−0.47 (0.002, 0.932)**
C-DII^TM^	0.29 (0.07)	**0.55 (<0.0001, 0.989)**	−0.09 (0.57)	−0.05 (0.77)	−0.29 (0.07)	0.30 (0.06)
Control group						
Fat, intake/1000 kcal	0.12 (0.48)	−0.15 (0.35)	−0.01 (0.95)	−0.06 (0.69)	−0.15 (0.37)	0.15 (0.35)
LA, % energy of diet	−0.18 (0.26)	0.01 (0.93)	−0.04 (0.80)	−0.16 (0.33)	−0.07 (0.66)	0.03 (0.86)
ALA, % energy of diet	−0.14 (0.38)	0.17 (0.29)	−0.08 (0.64)	0.19 (0.23)	−0.09 (0.60)	−0.09 (0.57)
PUFAs, % energy of diet	−0.22 (0.18)	−0.02 (0.91)	−0.07 (0.69)	−0.10 (0.55)	−0.07 (0.65)	−0.01 (0.95)
*n*−3 PUFAs, % energy of diet	−0.04 (0.83)	−0.22 (0.18)	−0.18 (0.27)	0.06 (0.71)	−0.23 (0.15)	0.13 (0.41)
EPA, mg/day	−0.14 (0.38)	−0.28 (0.082)	−0.24 (0.13)	0.31 (0.052)	−0.05 (0.75)	−0.09 (0.56)
DHA, mg/day	−0.05 (0.76)	−0.31 (0.05)	−0.10 (0.56)	0.01 (0.95)	0.03 (0.84)	−0.05 (0.76)
Fiber, intake/1000 kcal	0.13 (0.41)	**−0.31 (0.049, 0.561)**	−0.11 (0.50)	−0.04 (0.82)	0.20 (0.21)	−0.11 (0.50)
C-DII^TM^	0.24 (0.13)	0.20 (0.22)	0.05 (0.75)	−0.23 (0.15)	−0.25 (0.13)	**0.37 (0.018, 0.722)**

Data presented are Pearson’s correlation coefficient—r (*p*-value), correlations with *p* < 0.05 and post-hoc power, in bold. AA—arachidonic acid; ALA—α-linolenic acid; C-DIITM—energy-adjusted children’s dietary inflammatory index; DHA—docosahexaenoic acid; LA—linoleic acid; LC-PUFAs—long-chain PUFAs; PUFAs—polyunsaturated fatty acids; PUFA *n*-6/*n*-3—ratio of PUFAs from the *n*-6 to *n*-3 family.

## Data Availability

The data presented in this study are openly available in FigShare at https://doi.org/10.6084/m9.figshare.27948459.

## Abbreviations

AA: arachidonic acid; ALA, alpha-linolenic acid; BMI, body mass index; C-DII^TM^, Children’s Dietary Inflammatory Index; CVB, Coxsackievirus B; DHA, docosahexaenoic acid; DII^®^, Dietary Inflammatory Index; EPA, eicosapentaenoic acid; LA, linoleic acid; PUFAs, polyunsaturated fatty acids; RRIs, recurrent respiratory infections; specialized pro-resolving mediators, SPMs; VLC-PUFAs, very long chain polyunsaturated fatty acids.
